# A genome-wide association study identifies a possible role for cannabinoid signalling in the pathogenesis of diabetic kidney disease

**DOI:** 10.1038/s41598-023-31701-w

**Published:** 2023-03-22

**Authors:** Wael Osman, Mira Mousa, Mohammed Albreiki, Zahrah Baalfaqih, Hinda Daggag, Claire Hill, Amy Jayne McKnight, Alexander P. Maxwell, Habiba Al Safar

**Affiliations:** 1 Center for Biotechnology, Khalifa University, PO Box 127788, Abu Dhabi, United Arab Emirates; 2 Department of Biology, College of Arts and Sciences, Khalifa University, Abu Dhabi, United Arab Emirates; 3 Imperial College of London Diabetes Centre, Abu Dhabi, United Arab Emirates; 4 Centre for Public Health, Queen’s University of Belfast, Belfast, UK; 5 Department of Biomedical Engineering, College of Engineering, Khalifa University, Abu Dhabi, United Arab Emirates

**Keywords:** Genome-wide association studies, Clinical epigenetics, Genetic testing

## Abstract

Diabetic kidney disease (DKD), also known as diabetic nephropathy, is the leading cause of renal impairment and end-stage renal disease. Patients with diabetes are at risk for DKD because of poor control of their blood glucose, as well as nonmodifiable risk factors including age, ethnicity, and genetics. This genome-wide association study (GWAS) was conducted for the first time in the Emirati population to investigate possible genetic factors associated with the development and progression of DKD. We included data on 7,921,925 single nucleotide polymorphism (SNPs) in 258 cases of type 2 diabetes mellitus (T2DM) who developed DKD and 938 control subjects with T2DM who did not develop DKD. GWAS suggestive results (P < 1 × 10^–5^) were further replicated using summary statistics from three cohorts with T2DM-induced DKD (Bio Bank Japan data, UK Biobank, and FinnGen Project data) and T1DM-induced DKD (UK-ROI cohort data from Belfast, UK). When conducting a multiple linear regression model for gene-set analyses, the *CNR2* gene demonstrated genome-wide significance at 1.46 × 10^–6^. SNPs in *CNR2* gene, encodes cannabinoid receptor 2 or CB2, were replicated in Japanese samples with the leading SNP rs2501391 showing a *P*_combined_ = 9.3 × 10^–7^, and odds ratio = 0.67 in association with DKD associated with T2DM, but not with T1DM, without any significant association with T2DM itself. The allele frequencies of our cohort and those of the replication cohorts were in most cases markedly different. In addition, we replicated the association between rs1564939 in the *GLRA3* gene and DKD in T2DM (P = 0.016, odds ratio = 0.54 per allele C). Our findings suggest evidence that cannabinoid signalling may be involved in the development of DKD through *CB2*, which is expressed in different kidney regions and known to be involved in insulin resistance, inflammation, and the development of kidney fibrosis.

## Introduction

Type 2 diabetes mellitus (T2DM) is a chronic metabolic disorder associated with hyperglycemia due to a combination of insulin resistance and β-cell impairment, influenced by genetic and environmental factors^1^. Among the different complications that arise with T2DM, progressive loss of renal function represents one of the most serious complications, characterized as diabetic kidney disease (DKD), with an estimated prevalence of 30–50%^2^. DKD is a multifactorial, heterogeneous condition that is considered as the leading cause of progressive renal impairment and end-stage renal disease^3^. This condition results in pathologic changes associated with glomerular structure and function, albuminuria, excessive extracellular matrix deposition resulting in mesangial matrix expansion, inflammation, and fibrosis^4,5^. In addition to poor glycemic control, which can be controlled by pharmaceutical and clinical interventions, there also are several nonmodifiable risk factors associated with DKD, such as age, sex, ethnicity, and genetics^6^. Familial aggregation of DKD has been demonstrated in different ethnic groups, demonstrating the importance of understanding how environmental and genetic factors interact resulting in distinct disease predispositions and mechanisms in different populations^7–13^.

The Middle East region has some of the highest prevalence of T2DM worldwide, with a disproportionally high prevalence of DKD, ranging between 10.8 and 61.2%, rendering this particular cohort interesting for genetic studies of this trait^14,15^. Genetic and epigenetic studies of DKD, using candidate gene approaches and genome-wide studies, have been undertaken to identify genetic loci conferring susceptibility or resistance to DKD. Currently, more than 150 genes have been associated with DKD using candidate gene approaches, while about 42 genetic loci have been associated with DKD susceptibility or for DKD indicators, such as albuminuria, through genome-wide association studies (GWAS)^12,16–23^. There is, however, a few robustly replicated loci that have emerged, with a knowledge gap on the functional role of the newly identified genes in DKD, which would require further replication studies and detailed analysis in experimental models. Furthermore, there are a limited number of GWAS studies that have been conducted in the Middle Eastern populations related to T2DM-associated DKD.

Though DKD demonstrates familial aggregation and clustering, and SNP heritability, the genetic factors influencing DKD remain largely unknown^24–27^. To further explore the genetic association of DKD in the Middle Eastern population with T2DM, we performed a GWAS to identify possible genetic risk factors and uncover mechanisms that contribute to the development and progression of DKD among an Emirati population. Additionally, we aimed to replicate the strongest signals from the current analysis in different populations to confirm this association, as well as in the GWAS Catalog (European ancestry cohorts). We also aimed to replicate previously identified genome-wide significant associations in the Middle Eastern population.

## Methods

### Recruitment and ethical consideration

This cross-sectional study recruited 1223 unrelated Emirati participants from Imperial College of London Diabetic Centre (ICLDC), Abu Dhabi, United Arab Emirates, between the period of April 2018 to January 2020. The participants were divided into two clinical groups: T2DM with DKD (n = 261) and T2DM without DKD (n = 962). The diagnosis of T2DM was made by a qualified physician based on WHO guidelines and criteria, with an a HbA1c ≥ 6.5 and were receiving treatment for their condition, whereas DKD was diagnosed as outlined previously^28^. In summary, DKD was defined as either decreased estimated glomerular filtration rate (eGFR < 60 mL/min/1.73 m^2^) with or without renal damage over a period of at least 3 months, or based on an albumin-to-creatinine ratio ≥ 30 mg/g, or proteinuria > 500 mg over a 24-h period in the setting of T2DM and/or abnormalities, as assessed by imaging or histology^29^. The inclusion criteria were: (1) UAE nationals, (2) clinically diagnosed with T2DM with or without DKD, (3) above 18 years old and (4) able to provide informed consent and complete the survey. Participants were excluded if they were diagnosed with a critical condition (e.g., cancer) or pregnancy.

An informed consent was obtained from all participants. The study was conducted in accordance with the Declaration of Helsinki and has been approved by the Imperial College London Diabetes Centre (ICLDC) ethical committee (IREC 036).

### Pre-laboratory measurements

All participants completed a validated screening questionnaire that includes details on the demographic characterization, risk factors, symptomatology, and family history of diseases. Medical history, medication and management methods were provided by ICLDC. Anthropometric parameters and blood pressure were collected and assessed during the hospital visit by trained nurses using calibrated stadiometer, weight scale and sphygmomanometer. Biochemical data such as chemistry and hematology parameters, to assess the presence of diabetic complications, were obtained from ICLDC.

### DNA extraction, genotyping, imputation, and quality control

Total of 4 ml of blood sample was collected from each participant into EDTA anti-coagulant tubes. Genomic DNA was extracted using the automated MagPurix 24 system (LTF Labortechnik, GmbH&Co. KG, Hattnaeur, Wasserburg, Germany) according to the manufacturer’s instructions. DNA was quantified using DS-11 Series of Spectrophotometer/Fluorometer (DeNovix Inc. Wilmington, USA).

Genotyping was done using Infinium Global Screening Array (Illumina, Inc., San Diego, CA, USA), which contained 654,027 genetic markers and developed by Avera Institute for Human Genetics (South Dakota, USA). The Infinium Global Screening Array-24 v3.0 BeadChip would be best suited for this ethnically diverse cohort because it features a broad spectrum of diverse exonic content, including both cross population and population specific markers (EUR: 52,980 markers; EAS: 31,375 markers; AMR: 45,977 markers; AFR: 43,122 markers; SAS: 40,298 markers). Genotypes were exported, in Genome Reference Consortium human build 37 (GRCHb37) and Illumina ‘source’ strand orientation. The raw data was processed using Genome Studio (Version 2, Illumina, Inc.) genotyping module to generate the data for further analysis.

Stringent quality control measures were performed for samples and SNPs to ensure subsequent robust association tests. Samples that failed to reach a 98.0% call rate were reanalyzed. Samples were excluded if they had discordant sex information (n = 0) or outlying heterozygosity rate (n = 0). We also applied identity by state (IBS) and identity by descent (IBD) to exclude duplicated individuals or those who are first degree relatives (pairwise identity-by-state (PI_HAT) > 0.5, and accordingly, we excluded 27 individuals: three cases and 24 controls. Quality control measures for SNP callings were missing per individual (> 10%), missingness per marker (> 5%), minor allele frequency (MAF > 1%), Hardy–Weinberg Equilibrium (HWE > 0.00001). After quality control, a total of 443,465 variants passed filters.

Genotypes were prephased and imputed with untyped markers (∼39 M) using the Phase 3 multi-ethnic 1000 Genomes Projects panel, as the reference based on the human genome assembly hg19 (https://mathgen.stats.ox.ac.uk/impute/1000GP_Phase3.html), and was carried out by using BEAGLE, using standard protocols and recommended settings. Imputation was performed on both autosomal and sex chromosomes. Post imputation quality control measures (genotyping rate < 95%, call rate < 98%, MAF > 1%, HWE > 0.00001) were conducted, and a total of 7,921,925 variants passed filters.

### Statistical analysis

Continuous variables were presented as means and standard deviations, and categorical data were calculated as proportions and percentages. When needed, Kolmogorov–Smirnov test was used to test the normality of the data. student’s t-test (unpaired t-test) was used to test the significant differences of the pre-laboratory measurements between cases and controls. The significance level for pre-laboratory measurements was set at *p* < 0.05. These analyses were done using SPSS 22 (SPSS Inc., IBM Company, Chicago, Illinois, USA).

**Figure 1:**
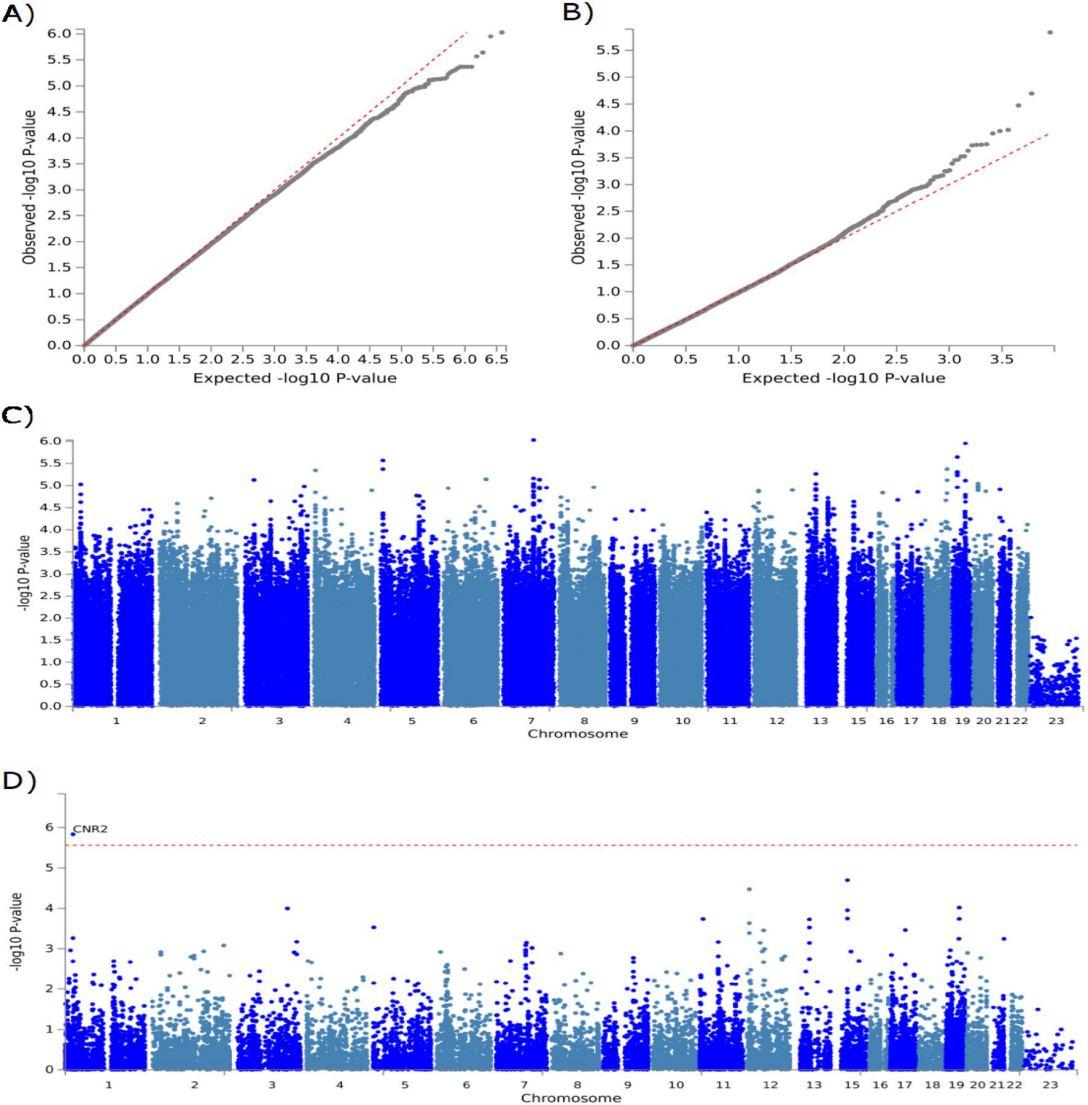
Q-Q plots and Manhattan plots of the DKD GWAS and gene-based test using FUMA and MAGMA. (**A**) Quantile–Quantile plot of the GWAS data. The observed p-values, on a log-10 scale, are plotted against their expected values under the null hypothesis assuming none of the sites have an effect. (**B**) Quantile–Quantile plot of the gene-based data. The observed p-values, on a log-10 scale, are plotted against their expected values under the null hypothesis assuming none of the sites have an effect. (**C**) Manhattan plot of the DKD GWAS. The plot shows the p-values on a log-10 scale (y-axis) by their chromosomal location (x-axis). (**D**) Manhattan plot of the DKD gene-based test as computed by MAGMA. The plot shows the p-values on a log-10 scale (y-axis) by their chromosomal location (x-axis). Input SNPs were mapped to 18,124 protein coding genes. Genome wide significance (Red dashed line in the plot) was defined at p = 0.05/18,124 = 2.759 × 10^–6^, labelling one gene (*CNR2*) that reached genome wide significance.

In the GWAS and replication analyses, we used a multivariate logistic regression model, assuming additive allelic effects for genotyped and imputed SNPs, to assess the statistical significance of each SNP with adjustment for age, gender, log BMI, and the first ten principal components as covariates. The significance levels for the GWAS were 6.3 × 10^−9^ (0.05/7,921,925) in the analysis following the Bonferroni correction for multiple testing. Odds ratios and 95% confidence intervals were calculated using the minor allele genotype as a reference. Principal component analysis (PCA) was performed using the case–control samples in this GWAS and a reference panel of populations in the 1000 Genome project (Fig. 1). Meta-analysis studies were performed using PLINK 1.09. Haploview v4·1 was used to conduct subsequent association analysis. Functional Mapping and Annotation of genetic associations (FUMA) was used to perform visualization Manhattan plots^30^. To assess the association of a given SNP with T2DM and DKD, the allelic frequencies were compared by means of a χ^2^ statistic, which yielded an individual p-value for each combination of SNP and allelic model. A quantile–quantile (Q-Q) plot analysis was carried out to check whether the distribution of the inflation *p*-values deviated from the expected distribution under the null hypothesis of no genetic association and investigate if the overall significance of the genome-wide associations is due to potential impact of population stratification. Systematic bias and the impact of population stratification was evaluated by calculating the genomic control inflation factor [λ _GC_] and noted for each analysis. A Manhattan plot was generated with −log_10_ *p*-values.

The gene-set analysis was conducted using the FUMA software, an online platform for functional mapping of genetic variants from GWAS summary^30^. The gene-set analyses was produced in Multi-marker Analysis of GenoMic Annotation (MAGMA), in which it examines sets of biologically related genes that are strongly associated to the disease of interest^31^. The MAGMA tool uses multiple linear regression models to assess whether genes in each gene set are associated to a polygenic trait, after correcting for linkage disequilibrium between variants and gene size.

### Replication analysis

Based on the top identified SNP in this cohort, further replication data was obtained from three datasets: DKD summary results of Biobank Japan^32^, the FinnGen Project^33^ (cohort name: finngen_R5_DM_NEPHROPATHY_EXMORE), and the UK Biobank^34^ (cohort name: E1122). The GWAS study for the Japanese population contains 220 cases with DKD vs. 132,764 controls. The Finnish samples included 3283 cases with DKD vs. 181,704 controls, and the UK Biobank contains 58 cases vs. 8444 controls. Replication analysis in type 1 diabetes-induced DKD was done in the UK-ROI cohort (Belfast, UK), white individuals with T1D, diagnosed before 31 years of age, whose parents and grandparents were born in the UK and Ireland^19^. The UK-ROI cohort case group comprised individuals with persistent proteinuria (> 500 mg/24 h) developing more than 10 years after the diagnosis of diabetes, hypertension (> 135/85 mmHg and/or treatment with antihypertensive medication), and retinopathy; ESRD (27.2%) was defined as individuals requiring renal replacement therapy or having received a kidney transplant. Absence of DKD was defined as persistent normal urine albumin excretion rate (AER; 2 out of 3 urine albumin to creatinine ratio [ACR] measurements, 20 mg of albumin/mg of creatinine) despite duration of T1D for at least 15 years, while not taking an antihypertensive medication, and having no history of treatment with ACE inhibitors. In the analysis in this study, there were 823 DKD/ESRD cases and 903 controls. DNA from individuals in the UK-ROI collection were genotyped using the Omni1-Quad array (Illumina, San Diego, CA, USA).

A replication analysis was conducted in this cohort on previously identified variants (SNPs and candidate genes) associated with DKD, totaling 49 SNPs in 42 gene loci. The direction of effect and strength of association was measured in this cohort.

## Results

### Study cohort characteristics

All study participants had T2DM, and both cases and controls (with or without DKD) demonstrated poor glycemic control indices (mean HbA1c > 7% for both groups). Moreover, those with DKD were mostly men (p < 0.001), older in age (p < 0.001), and had higher rates of associated comorbidities, such as hypertension (p < 0.001) and dyslipidemia (p < 0.001) (Table 1). The renal function (eGFR, p < 0.001; creatinine, p < 0.001), urea, p < 0.001; vitamin D, p = 0.002) and lipid profile (cholesterol total, p < 0.001; HDL, p = 0.002; LDL, p < 0.001) were significantly lower in participants with DKD than non-DKD patients. BMI demonstrated no significant association (p = 0.392) between cases and controls.

**Table 1:** Demographic data of the study participants.

Variable	Case (n = 258)	Control (n = 938)	P-value
Gender			
Male	188 (72.9%)	547 (58.3%)	< 0.001
Female	70 (27.1%)	391 (41.7%)	
Age (mean, SD)	64.81 (11.48)	53.95 (11.38)	< 0.001
Age (categories)			
< 46	19 (7.4%)	238 (25.4%)	< 0.001
47–54	30 (11.6%)	227 (24.2%)	
55–60	33 (12.8%)	211 (22.5%)	
61–67	67 (26.0%)	166 (17.7%)	
> 67	109 (42.2%)	96 (10.2%)	
BMI (mean, SD)	31.72 (6.40)	31.35 (5.92)	0.392
BMI (categories)			
< 18.50	1 (0.4%)	1 (0.1%)	0.480
18.51–24.49	24 (9.4%)	79 (8.5%)	
24.50–29.99	84 (32.9%)	346 (37.2%)	
> 30.00	146 (57.3%)	505 (54.2%)	
Hypertension			
No	36 (14.5%)	453 (52.0%)	< 0.001
Yes	212 (85.5%)	418 (48.0%)	
Dyslipidemia			
No	38 (15.2%)	234 (26.4%)	< 0.001
Yes	212 (84.8%)	654 (73.6%)	
Key glycemic indices			
Random glucose	8.18 (4.72)	7.93 (4.32)	0.433
HbA1c	7.31 (1.93)	7.01 (1.97)	0.039
Key renal function measures			
eGFR	60.14 (31.49)	109.93 (27.20)	< 0.001
Creatinine	139.70 (121.54)	64.81 (26.53)	< 0.001
Urea	9.44 (7.39)	4.23 (1.96)	< 0.001
Vitamin D	66.85 (44.79)	76.20 (43.25)	0.002
Lipid profile			
Cholesterol total	3.58 (1.17)	4.01 (1.25)	< 0.001
TG	1.49 (0.87)	1.51 (0.88)	0.702
HDL	1.13 (0.54)	1.24 (0.44)	0.002
LDL	1.94 (0.89)	2.43 (1.01)	< 0.001

eGFR: estimated glomerular filtration rate; HDL: high-density lipoprotein; LDL low-density lipoprotein; SD: standard deviation; TG: triglyceride.

### GWAS of DKD

To identify genetic variants that contribute to the susceptibility of DKD in the UAE population, we conducted a genome-wide association study (GWAS) using 258 cases with T2DM who developed DKD, and 938 control subjects who had T2DM but did not develop DKD. The samples were genotyped using the Illumina Infinium Global Screening Array (“Methods”). We selected 7,921,925 SNPs for further association analysis with DKD after applying stringent quality control (QC) filtering and imputation analyses (“Methods”). As shown in Supplementary Fig. 1, all cases and controls were representative with the structure of the UAE population as determined by PCA^35,36^. The full GWAS results of all suggestive loci are presented in the Supplementary Table 1. A Quantile–quantile (Q-Q) plot with the genomic control inflation factor [λ _GC_] of 1 indicated a low possibility of population stratification (Fig. 1A).

Based on the association analysis, twelve loci showed suggestive associations with DKD (p = 10^–5^, Fig. 1C and Supplementary Table 1). The leading SNPs of *PPP1R9A* gene, located in locus 7q21.3, was the most significant SNP at rs78595611 (p = 9.73 × 10^–7^) and SNP rs76361654 (p = 7.43 × 10^–6^). *PPP1R9A* gene is involved in neurite formation, is associated to coronary artery disease, and is a prognostic marker in renal cancer^37,38^. Two leading SNPs of the cytochrome P450 enzymes (*CYP2B7P*, rs4001941, p = 1.12 × 10^–6^; *CYP4F24P*, rs117074522, p = 2.27 × 10^–6^) that are involved in drug pharmacokinetics and response have also been associated with DKD in T2DM patients^39^. Gene *OR10H2* located in locus 19p13.12 and gene *STX18-AS1* in locus 4p16.2 are associated with acute myeloid leukemia and congenital heart disease, and the SNPs that have been associated to DKD are rs11664515 (p = 2.27 × 10^–6^) and rs16836018 (p = 2.27 × 10^–6^), respectively^40–42^. The *RB1* gene located at 13q14.2 has been associated with diabetic cardiomyopathy, chronic kidney disease, and proteinuria, all phenotypes that are associated with kidney function and DKD. The leading SNP of *RB1* gene is (p = 5.49 × 10^–6^)^43–45^. The expression of gene *MOXD1* located in locus 6q23.2 for SNP rs9493286 (p = 7.25 × 10^–6^) has been identified to be associated with diabetic nephropathy and obesity-related traits^46–48^.Gene *MACROD2* located in locus 20p12.1 have been associated with chronic kidney disease, hypertension, glomerular filtration rate, with the leading SNP rs11696648 demonstrating an association (p = 9.05 × 10^–6^)^41,49–51^. The *CNR2* gene in locus 1p36.1 (SNP rs542405361, p = 9.43 × 10^–6^) has been associated with diabetic nephropathy, kidney fibrosis and diabetes-induced cardiac dysfunction, all phenotypes associated with DKD^52–54^. Additionally, *CNR2* have been identified to alleviate inflammation, oxidative stress and fibrosis, suggesting an important role in kidney function and cardiovascular complications of diabetes^55^.

A gene-set analysis was conducted to identify genes that are associated with DKD, after implementing multiple regression model and multi-marker association. A Quantile–quantile (Q-Q) plot indicated low possibility of population stratification (Fig. 1B). When conducting a multiple linear regression model on the 18,124 protein coding genes, after correcting for linkage disequilibrium between the SNPs and gene size, the *CNR2* gene demonstrated genome-wide significance at 1.46 × 10^–6^, as demonstrated in Fig. 1D.

### Replication analyses—T2DM-induced DKD cohorts

SNPs with suggestive associations were selected for replication in three datasets: the DKD summary results for Biobank Japan^32^, the UK Biobank^34^, and FinnGen Project^33^. Consequently, SNPs in one locus, *CNR2*, were replicated in the Japanese samples, but not in the Finnish samples, with the leading SNP in rs2501391 in *CNR2* gene which we could replicate the Japanese samples with *P*_combined_ = 9.3 × 10^–7^, and OR = 0.67 (Table 2). The samples from FinnGen project showed very significant differences in allele frequencies in comparison to our samples as well as the Japanese samples (Table 2). The full replication analyses are presented in Supplementary Tables 1 to 4. It is interesting to note that rs2501391 was not the most prominent SNP associated with DKD in the *CNR2* gene. However, the more significant SNPs, such as rs542405361, were not found in the Japanese study to replicate them (Supplementary Tables 1 to 3). Due to the different ancestral background of the populations, this variant may be monomorphic in different ethnic groups. However, independent variants associated with DKD may be present in the same region for different ethnicities.

**Table 2:** GWAS and replication results of the SNP rs2501391 in *CRN2* gene with DKD in the UAE cohort and replication cohorts from Biobank Japan, UK Biobank, and FinnGen project.

SNP	Chr:BP	A1/A2	Cohort*	N (Case vs. Cont)	MAF		OR (95% CI)	SE	P	Meta-analysis		
					Case	Cont				P	OR	*P*_h_**
rs2501391	1:24,216,118	A/G	UAE	258/938	0.212	0.231	0.58 (0.32–0.84)	0.135	5.34 × 10^–5^			
			BBJ	220/132,764	0.393	0.321	0.73 (0.53–0.93)	0.101	1.92 × 10^–3^			
			UK-BB	58/8444	–	–	0.84 (0.48–1.47)		0.54			
			FinnGen	3283/181,704	0.002	0.001	0.99 (0.12–1.86)	0.443	0.75			
			UAE + BBJ							9.3 × 10^–7^	0.67	0.17
			UAE + UK-BB							7 × 10^–4^	1.51	0.022
			UAE + FinnGen							1.2 × 10^–4^	1.64	0.22
			All cohorts							7.6 × 10^–6^	1.41	0.099

A1: effective allele; BP: base pair, indicating chromosomal location; Chr: chromosome; CI: confidence intervals; Cont: controls; MAF: minor allele frequency; OR: odds ratio, SE: standard error.

*Cohorts: UAE: United Arab Emirates cohort, BBJ: Biobank Japan, UK-BB: UK Biobank, FinnGen: Finnish cohort.

**P_h_: p-value for heterogeneity of Cochrane's Q statistic.

### Replication analyses—T1DM-induced DKD cohorts

To test for the existence of a shared pathway for DKD in both type 1 and type 2 diabetes, SNPs with suggestive associations were also selected for replication in replication analysis in type 1 diabetes-induced DKD in the UK-ROI cohort from Belfast, UK. This is a cohort of 823 DKD/ESRD cases and 903 controls of white individuals from Ireland and the UK (see “Methods”). As shown in Table 3, none of the SNPs showed significant associations with GWAS SNPs. The lead SNP in the *CNR2* gene, rs2501391, was not available in the UK-ROI cohort; however, it is highly linked to SNP rs2502959 (r2 = 0.98, D′ = 1), suggesting no significant association with T1DM-induced DKD.

**Table 3:** Replication analysis of the suggestive GWAS loci with T2DM in UK-ROI cohort from Belfast, UK.

SNP	Chr	Gene	A1	SE	OR (95% CI)	P-value
rs4001941	19	*CYP2B7P*	A	0.091	0.97 (0.81–1.16)	0.73
rs11696648	20	*MACROD2*	A	0.098	1.17 (0.97–1.42)	0.11
rs2502959	1	*CNR2*	A	0.108	0.98 (0.8–1.22)	0.89
rs2033958	4	*STX18-AS1*	C	0.083	1.0 (0.85–1.17)	0.96
rs6853673	4	*STX18-AS1*	T	0.084	0.99 (0.84–1.16)	0.88
rs1510798	4	*STX18-AS1*	T	0.084	1.0 (0.85–1.18)	0.98
rs188011	5	*RP11-332J15.1*	T	0.098	1.0 (0.82–1.21)	0.98
rs1548395	7	*PPP1R3A*	T	0.086	0.93 (0.79–1.1)	0.41
rs2968844	7	*PPP1R3A*	T	0.084	0.91 (0.77–1.07)	0.25
rs2023691	7	*PPP1R3A*	C	0.085	0.95 (0.81–1.12)	0.56
rs1119660	7	*PPP1R3A*	C	0.095	0.86 (0.71–1.03)	0.11
rs11534004	7	*PPP1R3A*	G	0.095	0.86 (0.71–1.04)	0.11
rs12605711	18	*LOC107985179*	T	0.102	1.02 (0.83–1.24)	0.88
rs6135660	20	*MACROD2*	T	0.089	1.1 (0.92–1.30)	0.31
rs1575443	20	*MACROD2*	G	0.098	1.15 (0.95–1.4)	0.15

A1: effective allele; Chr: chromosome; CI: confidence intervals; Cont: controls; MAF: minor allele frequency; OR: odds ratio, SE: standard error.

### Association of *CNR2*-rs2501391 with T2DM

We further investigated whether the *CNR2*-rs2501391 polymorphism is associated with T2DM itself. A genotype analysis was performed on this SNP in a cohort of 364 patients with T2DM and 103 healthy controls in an independent cohort. In the association analysis, we found that this SNP had no association with T2DM (Table 4), which suggests that *CNR2* may be contributing to DKD development, and not T2DM per se.

**Table 4:** Association of *CNR2*-rs2501391 with T2DM in the UAE population.

	Descriptive statistics			Regression analysis			
Genotype	Diabetes (%)	Non-diabetes (%)	P-value	Crude OR (95% CI)	P-value	Adjusted OR* (95% CI)	P-value
AA	285 (78.3%)	86 (83.5%)	0.332	1.00		1.00	
AG	67 (18.4%)	16 (15.5%)		0.27 (0.04–2.15)	0.220	1.14 (0.42–3.14)	0.792
GG	12 (3.3%)	1 (1.0%)		0.35 (0.04–2.88)	0.349	3.81 (0.32–46.10)	0.293

*Multivariate logistic regression adjusted for age, gender and logBMI.

### Replication analysis of previous studies on DKD

We also replicated previous associations with DKD or kidney function indicators of DKD from previous GWAS that collectively reported 49 SNPs in 42 gene loci^12,17,19–23,56^. Based on our dataset, we evaluated the associations of 16 SNPs, and we were only able to replicate the association of rs1564939 in the *GLRA3* gene with DKD (p = 0.016, OR = 0.54 per allele C, Table 5). In most cases, there were marked differences between the allele frequencies of our cohort and those of the original cohorts at these SNPs.

**Table 5:** Association of previous loci reported with DKD and kidney function in diabetic patients in the UAE cohort.

SNP	Chr:BP	Gene	A1	MAF	MAF*	P	OR (95% CI)	OR*	Ref
rs12615970	2:3,745,215	*COLEC11, ALLC*	G	0.066	0.133	0.355	1.21 (1.2–1.23)	0.77	^17,18^
rs7583877	2:100,460,654	*AFF3*	C	0.453	0.289	0.109	1.2 (1.2–1.21)	1.29	^19^
rs4972593	2:174,462,854	*Sp3*	A	0.094	0.14	0.647	1.08 (1.07–1.1)	1.81	^21^
rs7588550	2:213,168,768	*ERBB4*	G	0.011	0.052	0.132	1.92 (1.86–1.97)	0.66	^19^
rs55703767	2:228,121,101	*COL4A3, COL4A4*	T	0.057	0.206	0.150	1.38 (1.36–1.4)	0.78	^17,18^
rs6436688	2:228,259,302	*COL4A3*	A	0.397	0.56	0.398	0.91 (0.9–0.92)	1.13	^18^
rs1564939	4:175,651,499	*GLRA3, GLRA4*	C	0.046	0.18	0.016	0.54 (0.53–0.55)	–	^20,22^
rs10011025	4:175,654,223	*GLRA3*	G	0.045	0.16	0.073	0.64 (0.63–0.65)	–	^20,22^
rs12509729	4:175,655,143	*GLRA3*	A	0.031	0.16	0.136	0.65 (0.63–0.66)	–	^20^
rs118124843	6:30,887,465	*DDR1, VARS2*	T	0.051	0.011	0.065	0.52 (0.51–0.53)	3.99	^17^
rs9942471	6:89,948,232	*GABRR1*	C	0.167	0.64	0.194	1.2 (1.19–1.21)	1.15	^12^
rs2410601	8:18,922,577	*PSD3, SH2D4A*	C	0.473	–	0.176	0.86 (0.86–0.87)		^22^
rs551191707	8:128,100,029	*PRNCR1*	CA	0.036	0.122	0.950	1.02 (1–1.04)	1.71	^17,18^
rs12437854	15:94,141,833	*RGMA, MCTP2*	G	0.029	0.038	0.361	0.76 (0.75–0.77)	1.8	^19^
rs56094641	16:53,806,453	*FTO*	G	0.356	0.252	0.08	1.22 (1.21–1.23)	1.23	^23^
rs2206136	20:9,351,150	*PLCB4*	A	0.352	0.42	0.198	0.86 (0.85–0.86)	1.12	^12^

A1: effective allele; BP: base pair, indicating chromosomal location; Chr: chromosome; CI: confidence intervals; MAF: minor allele frequency of the UAE cohort; MAF*: minor alleles frequency according to the reference study; OR: odds ratio; OR*: odds ratio according to the reference study; Ref: references.

## Discussions

We identified a gene-based genome-wide significant variant in *CNR2* gene*,* located in 1p36.11, which encodes the cannabinoid receptor 2 (CB2), associated with DKD in an Emirati cohort. After a replication and meta-analysis of GWAS variants across ancestry groups, we identified a similar signal in the Japanese population, but no association in the Finnish or UK-ROI population. We have discovered an additional eleven variants (rs78595611 in *PPP1R9A* gene; rs4001941 in*CYP2B7P*; rs117074522 in*CYP4F24P*; rs275475 in *RP11-332J15.1*; rs11664515 in *OR10H2*; rs16836018 in*STX18-AS1*; rs17349061 in*RB1*; rs9493286 in*MOXD1*; rs76361654 in *PPP1R3A*; rs141291445 in *LINC00693*; and rs11696648 in*MACROD2*) with suggestive association of DKD that will need to be replicated in a larger cohort, across multiple ancestral ethnic groups from Middle East populations.

When investigating non-genetic effects, several different factors contribute to the development of DKD in patients with T2DM, including hypertension and dyslipidemia with elevated cholesterol level, HDL, and LDL. These factors are reflected in this study and are independently associated with progressive DKD through lipid accumulation in tubular epithelial cells of diabetic kidneys^57^. A decline in renal function, urea, and vitamin D were also evident in those with DKD. Those observations are in line with previous study findings^28,58–61^. The presence of hyperglycemia initiates and sustains pathogenic pathways in the kidney that lead to the development and progression of DKD, but several other factors enhance this process even further. This is enhanced by several different factors that will facilitate the progression of the condition. Through optimized diabetes care, implementing pragmatic guidelines for therapeutic treatment, and appropriate targeting of education and support, the prognosis of patients with DKD may be dramatically improved^62,63^.

The *CNR2* gene, encoded by CB2, demonstrated genome-wide significance (p = 1.46 × 10^–6^) at a gene-set level, and has been implicated in mediating anti-inflammatory effects and immunomodulatory potential^64,65^. GWAS have associated variations in the *CNR2* gene with blood inflammatory cells, especially eosinophils and lymphocytes^54,66,67^. CB2 receptors may also regulate insulin secretion, insulin resistance, obesity-related inflammation, and metabolic changes such as nonalcoholic fatty liver disease^68,69^. In mouse and rat models, endocannabinoids generated in renal cells have demonstrated oxidative stress, inflammation, and renal fibrosis^70^. While there are conflicting reports regarding *CB2*'s role in DKD development^71,72^, recent studies suggest that *CB2* is upregulated by ischemia–reperfusion injury in mice and patients, which is associated to progressive kidney fibrosis^73^. Additionally, Zhou et al. demonstrated that genetic ablation and selective antagonists inhibiting CB2 were protective against kidney fibrosis, suggesting that CB2 plays an important role in kidney fibrosis pathogenesis^74^. This suggests that endocannabinoids through CB2 may actually play a dual conflicting role in fibrosis in T2DM. CB2 receptor-mediated responses contribute to both age-related and diet-induced insulin resistance, suggesting that these receptors may be useful therapeutic targets not only for kidney fibrosis, but also for obesity and diabetes as well^75^. Several factors, such as downstream cellular signaling, activation of b-catenin pathway, type and timing of kidney injury, and genetic variations in *CNR2* gene, particularly among different ethnic groups, may influence the receptor function and lead to the various pathological changes observed in the kidney during DKD.

Replication results of previously reported loci that were associated with DKD in patients with T2DM was conducted. SNP rs1564939 in *GLRA3* was significant (p = 0.016). This SNP has been associated with albuminuria, an early sign of DKD, in several publications conducted in European populations^20^. The *GLRA3* plays a major role in the central nervous system and has also been linked to ischemic damage. Glycine may also increase effective renal plasma flow and GFR, as well as decrease proximal and distal tubular sodium reabsorption by increasing renal interstitial hydrostatic pressure^76^. The remaining genetic locus were not replicated in the Middle Eastern cohort. This may be due to ascertainment and selection bias across the studies, or the differences in genetic structure, as indicated by the marked differences in allele frequencies, in which the study was conducted. Emerging evidence suggests that individuals of Middle Eastern descent have a different genome structure due to consanguineous marriage, endogamous unions, tribal structure, and larger family sizes^77^; however, genome studies from ethnic groups from the region are scarce, representing only 0.08% of genome data in the public domain^78^. Therefore, a larger cohort study of Middle Eastern descent must replicate these previously reported loci to attribute the lack of association.

There are several limitations in our study. The power analysis indicated that the sample size is barely sufficient to identify genome-wide significant variants. However, regardless of this, after conducting a gene-set analyses on the GWAS data, we were able to identify a genome-wide significant marker associated to DKD. The association of *CNR2* gene should be replicated in a larger, multi-ethnic cohort that would be more suited to investigate the genetic association, functional enrichment analysis and functional validation. Due to the lack of information available in this study to adjust for duration of diabetes, future studies should adjust for this confounding variable as it may serve as a genetic risk factor. While presentation, phenotypic profile and clinical course of DKD may be heterogeneous, the recruitment was conducted from one center to avoid ascertainment bias and limit misclassification in the case group. However, the heterogeneous nature of DKD, as well as differences in research design, method, ethnicity and gender compositions of the cohorts in the included studies, may have impacted the replication analysis. Future studies must be conducted in a larger cohort, with subgroup analysis and sensitivity analysis of the different phenotypic stages of DKD. Additionally, due to the lack of comparable studies and datasets available for DKD, we were unable to replicate our GWAS results in independent cohorts from the region; furthermore, we were unable to provide functional studies for the polymorphisms described in this study due to the absence of tissues and biopsies from patients. It is important to note that the replication analysis was performed on samples obtained from biobanks that employ different types of controls than were used in this study. According to the FinnGen or BBJ flow of studies, individuals with a specific phenotype or endpoint are treated as cases, while all other individuals without the phenotype are treated as controls, explaining the large number of controls compared to cases. Our definition of controls was therefore much narrower than in these cases, which may have resulted in a failure of replication of some signals in addition to the differences in genetic structure. Another limitation of this study is that DKD markers such as rs2501391 did not show an association with T2DM risk, or with a previously identified DKD loci. Due to the limited sample size and power, even though this might suggest that genes such as *CB2* could contribute to DKD development, it does not exclude the possibility that they may also confer risk for T2DM itself. Lastly, the use of a Euro-centric GWAS array limits the possibility of detected targeted SNPs in the genome that is ethnic-specific^79–83^. However, to limit this error, the imputation of the genotypes increases the detection of genetic markers that were not directly genotypes and provides further information on the association of these markers, and their respective haplotypes, to the condition.

In summary, we have discovered eleven variants that may be associated with DKD pathogenesis in T2DM patients. However, a highly plausible and significant association was identified in *CNR2* gene, suggesting that endocannabinoids signalling through CB2 receptor may be associated with DKD pathogenesis. Further functional studies are warranted to establish the role of *CNR2* gene, to improve fundamental knowledge and underlying biological pathway of DKD heterogeneous phenotypes. Performing GWAS studies across different racial and ethnic populations allow the identification of genes and haplotypes associated with different clinical outcomes of DKD. Further studies must be conducted on a large-scale, multi-ethnic cohort to substantiate our current knowledge on DKD pathogenesis and facilitate the development of population-specific therapeutic advances.

## Supplementary Information

Supplementary Figure 1.

Supplementary Tables.

## Data Availability

Access to raw data supporting the conclusions of this article will be made available by the corresponding author Dr Habiba Alsafar.
