# Supplementary material for: A genome-wide association study identifies a possible role for cannabinoid signalling in the pathogenesis of diabetic kidney disease
Source: Sci Rep. 2023 Mar 22;13:4661. doi: 10.1038/s41598-023-31701-w (PMC10033677; doi:10.1038/s41598-023-31701-w)
Supplement: Supplementary file 1 — Supplementary Figure 1. [file 41598_2023_31701_MOESM1_ESM.docx]

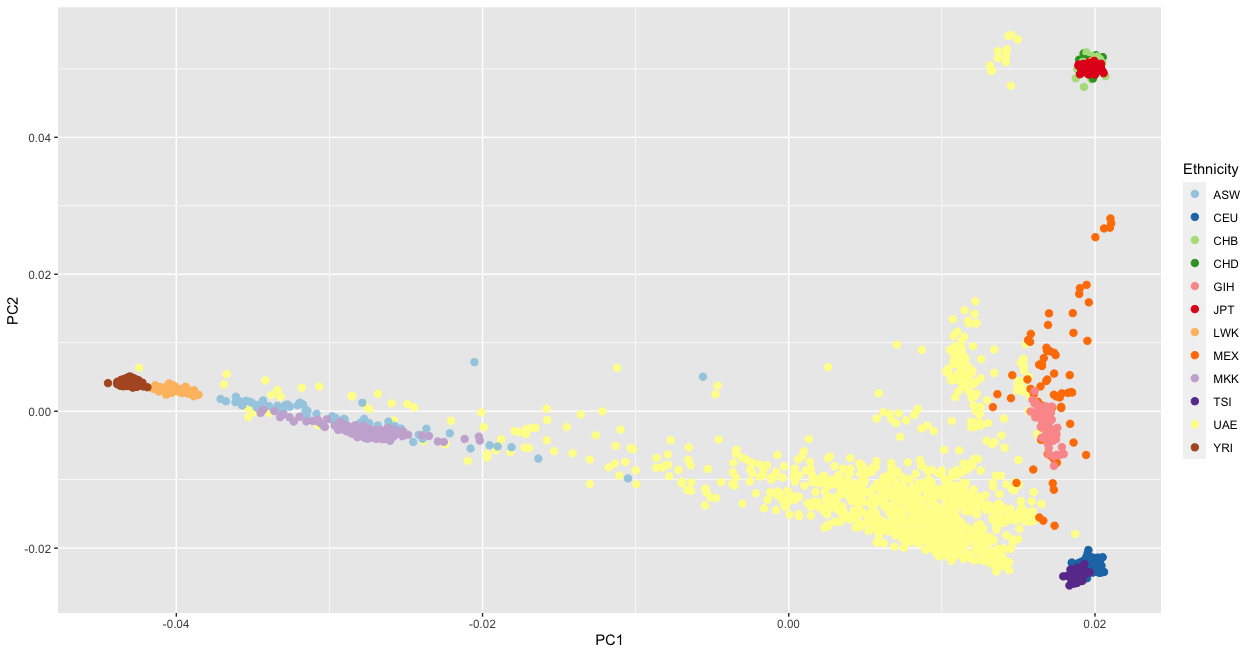


**Supplementary Figure 1. Admixture informed principal component analysis (PCA) plot of 1,237 participants from the UAE cohort and 1,092 samples from the 1000 Genome Project v3.** This cohort (defined as UAE in the PCA plot) is represented as yellow, demonstrating a diverse genetic pool. The population samples that were included in the PCA plot are: ASW (African ancestry in Southwest USA), CEU (Utah residents with Northern and Western European ancestry with CEPH collection), CHB (Han Chinese in Beijing, China), CHD (Chinese in Metropolitan Denver, Colorado), CLM (Colombian in Medellin, Colombia), FIN (Finnish in Finland), GBR (British from England and Scotland), IBS (Iberian Populations in Spain), JPT (Japanese in Tokyo, Japan), LWK (Luhya in Webeye, Kenya), MXL (Mexican ancestry in Los Angeles, California), PUR (Puerto Rican in Puerto Rico), TSI (Tuscany in Italia), UAE (United Arab Emirates cohort), YRI (Yoruba in Ibadan, Nigeria).
